# Extreme Airway Difficulty During EXIT in Severe Fetal Agnathia: Failed Conventional Intubation and Successful Emergency Tracheostomy—A Case Report

**DOI:** 10.1155/cria/9422230

**Published:** 2026-08-23

**Authors:** Rayan Muawad, Abdullah AlDhuwaihy, Hamad Alzomaia, Amna Kashgari, Abdullah Al Hammad, Mohammed Halawani, Jawad Alhabeeb, Mostafa Nagy

**Affiliations:** ^1^ Department of Pediatric Anesthesia, King Abdullah Specialized Children Hospital, MNGHA, Riyadh, Saudi Arabia; ^2^ College of Medicine, King Saud University, Riyadh, Saudi Arabia, ksu.edu.sa; ^3^ Medical Imaging Department, King Abdullah Specialized Children Hospital, MNGHA, Riyadh, Saudi Arabia; ^4^ Otolaryngology Division, King Abdullah Specialized Children Hospital, MNGHA, Riyadh, Saudi Arabia; ^5^ College of Medicine, King Saud bin Abdulaziz University for Health Sciences, Riyadh, Saudi Arabia, ksau-hs.edu.sa; ^6^ Department of Anesthesia and Critical Care Medicine, Cairo University, Cairo, Egypt, cu.edu.eg

**Keywords:** agnathia, EXIT procedure, fetal airway obstruction, micrognathia, prenatal MRI, tracheostomy, virtual bronchoscopy

## Abstract

**Background:**

Severe mandibular hypoplasia, particularly agnathia, is a rare congenital condition that may cause critical neonatal airway obstruction. The Ex Utero Intrapartum Treatment (EXIT) procedure allows airway control while maintaining uteroplacental circulation; overall airway establishment success rates for EXIT exceed 90%, yet conventional endotracheal intubation fails in a meaningful subset of cases with extreme anatomic distortion, necessitating surgical airway access. In the most severe forms of agnathia, primary tracheostomy under placental support may represent the most appropriate airway strategy from the outset.

**Case Presentation:**

A 36‐year‐old gravida 5 para 4 woman was referred after prenatal ultrasound detected severe fetal facial abnormalities. Serial imaging demonstrated progressive micrognathia evolving toward agnathia. Prenatal ultrasound demonstrated a markedly abnormal inferior facial angle (IFA) of 11° (normal mean 65° ± 16°) and a fetal nasomental angle of 92.87° (normal approximately 147°), indicating profound mandibular hypoplasia. Fetal MRI performed at 35 weeks and 3 days confirmed these findings, demonstrating an IFA of 40° and a nasomental angle of 116°, both below normal thresholds. The pregnancy was complicated by severe polyhydramnios. Fetal magnetic resonance imaging confirmed near‐complete absence of the mandible with glossoptosis and severe narrowing of the oropharynx and hypopharynx, while the distal trachea and bronchi appeared normal. Because of the anticipated risk of airway obstruction, a multidisciplinary team planned an EXIT‐to‐airway procedure at late preterm gestation. The mother underwent general anesthesia with rapid sequence induction using propofol and suxamethonium. Uterine relaxation was achieved via inhaled sevoflurane at 1.2 MAC and nitroglycerin infusion at 0.5 mcg/kg/min. Fetal heart rate was continuously monitored using a pulse oximeter applied to the fetal foot after partial delivery. During the procedure, attempts at airway establishment using video laryngoscopy and rigid bronchoscopy failed due to the inability to identify a recognizable epiglottis or glottic opening. The decision to proceed with emergency tracheostomy was made after three unsuccessful video laryngoscopy attempts, during which the airway appeared completely obliterated with no visible laryngeal inlet. Mask ventilation was also ineffective because of absent mandibular support and severe upper airway obstruction. An emergency tracheostomy was, therefore, performed while uteroplacental circulation was maintained, resulting in successful ventilation and stabilization of the neonate.

**Conclusion:**

This case highlights the limitations of conventional intubation during EXIT in fetuses with extreme mandibular deficiency. Detailed prenatal imaging is essential for anticipating airway challenges. In cases with complete upper airway obliteration, planned primary tracheostomy under placental support should be considered the primary airway strategy rather than a rescue measure.

## 1. Introduction

Severe mandibular hypoplasia, particularly agnathia (near‐complete or complete absence of the mandible), is strongly associated with profound upper airway obstruction at birth [1, 2]. When extreme micrognathia or severe glossoptosis (posterior displacement of the tongue) is identified antenatally, the Ex Utero Intrapartum Treatment (EXIT)‐to‐airway procedure is recommended. This procedure allows the fetal airway to be secured while uteroplacental gas exchange is maintained [1–3].

EXIT has become an established option for fetuses with anticipated airway compromise due to conditions like micrognathia, large cervical masses, or congenital high airway obstruction syndrome (CHAOS) [1, 2]. However, the EXIT procedure does not guarantee successful intubation. In the most severe forms of micrognathia and agnathia, the resulting anatomic distortion can prevent visualization of the laryngeal inlet, even with advanced tools such as video laryngoscopy or rigid bronchoscopy [1–3].

Accurately predicting which fetuses will fail conventional intubation during the EXIT procedure remains challenging, yet it has major implications for perinatal counseling and multidisciplinary team preparation [3, 4]. Recent advances in fetal imaging, including refined biometric indices of mandibular hypoplasia and dynamic MRI, have improved the antenatal recognition of fetuses at high risk for a difficult neonatal airway [3, 4]. Moving beyond conventional 2D imaging, newer techniques like 3D MRI airway reconstruction and virtual fetal bronchoscopy allow for volumetric visualization of the pharyngeal and laryngeal lumen, potentially offering superior prediction of airway feasibility [5–8]. Virtual bronchoscopy, derived from MRI data, has been shown to successfully delineate airway patency in fetuses with obstructive lesions, correlating well with postnatal findings [6, 7].

We report on a fetus with extreme micrognathia/agnathia, severe narrowing of the oropharynx and hypopharynx, and polyhydramnios in whom conventional intubation failed during the planned EXIT‐to‐airway procedure despite the use of video laryngoscopy and rigid bronchoscopy, ultimately necessitating an emergency tracheostomy. This case integrates detailed ultrasound and MRI findings and illustrates how advanced 3D MRI airway modeling and virtual bronchoscopy might have predicted conventional intubation failure and supported a planned primary tracheostomy [6–8].

## 2. Case Presentation

**FIGURE 1:**
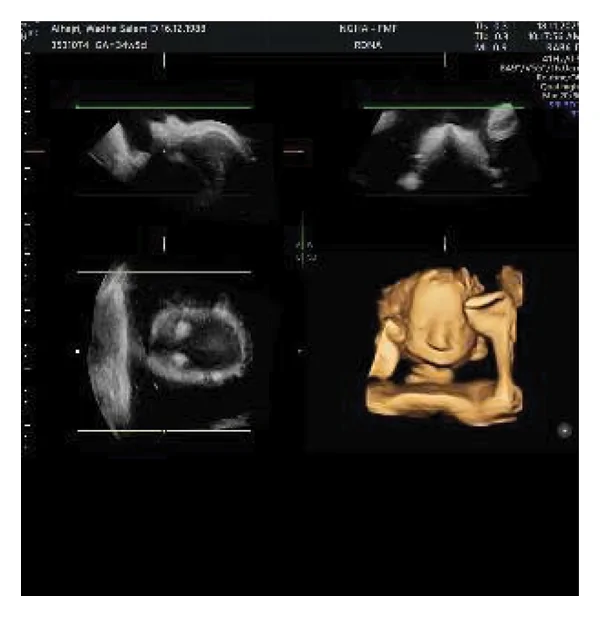
Prenatal three‐dimensional ultrasound imaging of severe micrognathia/agnathia.

**FIGURE 2:**
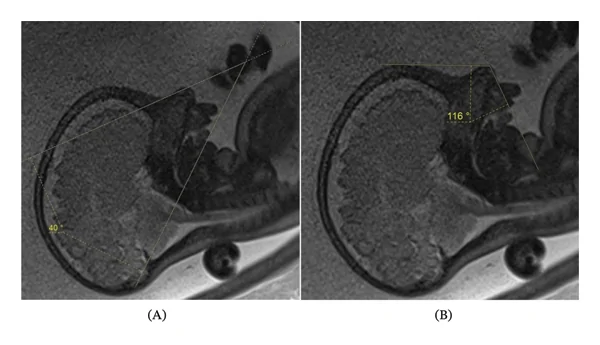
Prenatal biometric measurements of mandibular hypoplasia. (A) Fetal MRI demonstrating the inferior facial angle (IFA) of 40°, below the normal threshold of > 49°, consistent with severe micrognathia. (B) Sagittal T2‐weighted fetal MRI demonstrating the nasomental angle of 116°, significantly below the normal value of approximately 147°, further confirming severe mandibular deficiency.

A 36‐year‐old gravida 5 para 4 was referred to a tertiary fetal medicine center after a routine obstetric ultrasound detected abnormal fetal facial anatomy. Serial ultrasound examinations confirmed severe micrognathia that progressed toward agnathia (Figure 1). Biometric assessment on ultrasound demonstrated an inferior facial angle (IFA) of 11° (normal mean 65° ± 16°) and a fetal nasomental (FNM) angle of 92.87° (normal approximately 147° ± 2.7°), both markedly abnormal and consistent with profound mandibular hypoplasia. Subsequent fetal MRI confirmed these findings, demonstrating an IFA of 40° and a nasomental angle of 116° on sagittal imaging (Figure 2A,B), both below normal thresholds, reflecting severe mandibular deficiency. Due to the near‐complete absence of the bony mentum, the mandibular body could not be reliably measured. Visualization of the tongue was limited, and glossoptosis was suspected. The fetus developed severe polyhydramnios, with an amniotic fluid index ranging from 32 to 41.9 cm, which was attributed to impaired fetal swallowing.

Aside from a transient abdominal circumference at the 1st percentile, fetal biometry remained appropriate for gestational age, and umbilical artery Doppler indices were normal. No additional structural anomalies were identified in the fetal heart, thoracic structures, spine, limbs, or abdominal organs. These findings strongly suggested isolated, extreme micrognathia/agnathia with a very high risk of postnatal airway obstruction. Fetal MRI was performed at 35 weeks and 3 days of gestation for a more detailed characterization of the airway and associated structures.

**FIGURE 3:**
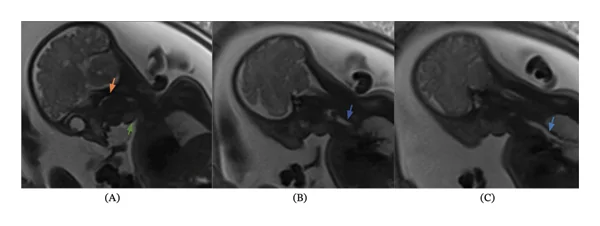
Sagittal T2‐weighted MRI images. (A) Severe micrognathia/agnathia with retrognathia (green arrow), severe narrowing/obliteration of the oropharynx and hypopharynx, and glossoptosis with posterior displacement of the tongue toward the pharyngeal wall (red arrow). The distal airways remain fluid filled (blue arrow). (B, C) Normal fluid‐filled distal trachea and bronchi, effectively excluding congenital high airway obstruction syndrome (CHAOS).

MRI confirmed severe micrognathia/agnathia with marked retrognathia and demonstrated severe narrowing or apparent obliteration of the oropharynx and hypopharynx (Figure 3A). Glossoptosis was evident, with the tongue displaced posteriorly toward the posterior pharyngeal wall (Figure 3A, red arrow). The distal trachea and bronchi appeared normal and fluid filled (Figure 3B,C), effectively excluding CHAOS. The nasal cavities, brain, and other major organs appeared structurally normal.

**FIGURE 4:**
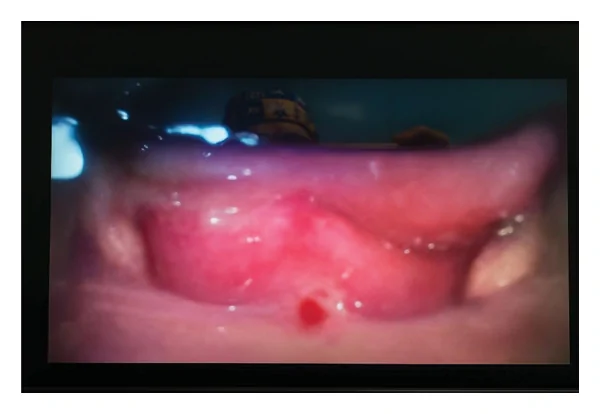
Intraoperative endoscopic image obtained with video‐laryngoscopy showing grossly distorted upper airway anatomy with complete inability to identify the glottic opening.

**FIGURE 5:**
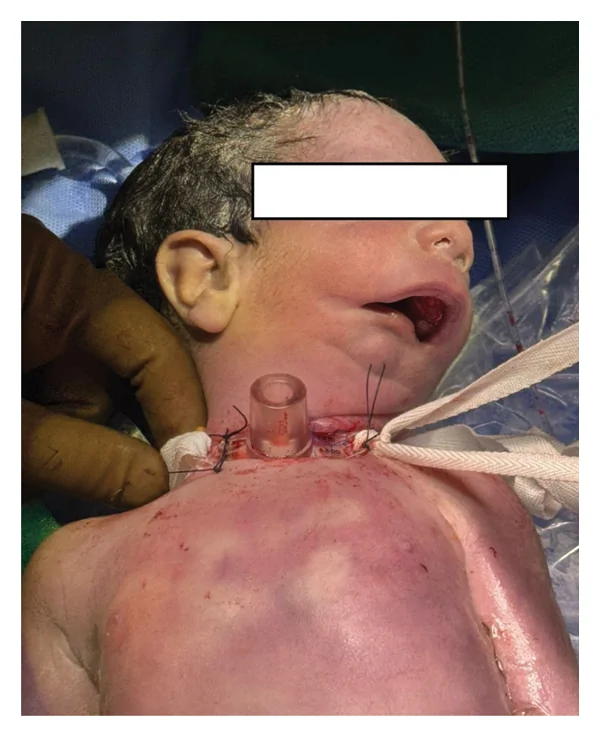
Postnatal clinical image demonstrating severe mandibular deficiency (agnathia) and the presence of the tracheostomy tube.

The overall impression was isolated, extreme mandibular deficiency leading to upper airway obstruction at the level of the oropharynx–hypopharynx. Given the high anticipation of airway difficulty, a multidisciplinary team—including obstetrics, neonatology, anesthesiology, pediatric otolaryngology, and pediatric surgery—planned an EXIT‐to‐airway procedure at 35 weeks and 5 days of gestation. The neonate was delivered with a birth weight of 2.5 kg. Under general anesthesia with uterine relaxation, a low transverse hysterotomy was performed, and the fetal head, neck, and upper torso were partially delivered while maintaining uteroplacental circulation. The interval from hysterotomy to umbilical cord clamping was approximately 25 min. Estimated maternal blood loss was 700 mL, with no significant intraoperative adverse events. Initial airway management was attempted via video laryngoscopy by the pediatric anesthesiologist. Despite optimal head positioning and repeated gentle attempts, the epiglottis and glottic opening could not be visualized. The extreme mandibular deficiency prevented effective displacement of the tongue, and only a blind mass of soft tissue was seen (Figure 4). The pediatric otolaryngologist then performed rigid bronchoscopy. The oropharyngeal anatomy was found to be grossly distorted, and no recognizable laryngeal inlet could be identified. Attempts to advance the scope into the airway were unsuccessful. Subsequently, bag‐mask ventilation proved ineffective due to the absent mandibular support and severe upper airway obstruction. In light of the failed intubation and ineffective mask ventilation, an emergency tracheostomy was performed while uteroplacental circulation was maintained. A transverse cervical incision was made, the trachea was identified, and a 3.3–3.5 mm tracheostomy tube was successfully inserted. Ventilation through the tracheostomy resulted in immediate and sustained improvement in chest movement and oxygenation. After confirming stable ventilation and hemodynamics, the umbilical cord was clamped, and the neonate was transferred to the neonatal intensive care unit with the tracheostomy in situ (Figure 5). In the neonatal period, the infant remained hemodynamically stable on mechanical ventilation via the tracheostomy. The NICU course was complicated by bilateral pneumothorax requiring chest tube insertion, which subsequently resolved. Cranial ultrasound and neurological assessment demonstrated no evidence of hypoxic–ischemic injury. The infant was discharged after 12 weeks with the tracheostomy in situ, pending further reconstructive evaluation (Table 1).

**TABLE 1:** Timeline of clinical events.

Gestational age/clinical phase	Diagnostic finding/intervention	Clinical outcome/status
Routine obstetric screening	Ultrasound identifies abnormal fetal facial anatomy, severe polyhydramnios (AFI 32–41.9 cm).	Referral to tertiary fetal medicine center.
35 weeks, 3 days gestation	High‐resolution fetal MRI confirms severe agnathia (Ultrasound IFA: 11°, MRI IFA: 40°; FNM angle: 116°), severe retrognathia, and oropharyngeal/hypopharyngeal obliteration.	Distal airways noted to be fluid filled, excluding CHAOS. Multidisciplinary planning initiated.
35 weeks, 5 days gestation	Execution of EXIT‐to‐airway procedure under maternal GA (sevoflurane + nitroglycerin infusion).	Continuous fetal foot pulse oximetry monitoring established under placental support.
EXIT procedure (intraoperative)	3 separate conventional airway visualization attempts (video laryngoscopy + rigid bronchoscopy) reveal completely blind tissue mass with no identifiable laryngeal inlet.	Decision made to halt conventional approaches due to absolute anatomic obstruction.
EXIT procedure (surgical airway)	Rapid conversion to emergency open tracheostomy (3.3–3.5 mm tube inserted). Total time from hysterotomy to cord clamp: 25 min.	Successful bilateral lung expansion and immediate oxygenation stabilization under placental support. Cord clamped.
Postnatal NICU phase (Weeks 1‐2)	Neonate stabilized on mechanical ventilation. Course complicated by bilateral pneumothorax.	Chest tubes placed with complete resolution. Normal cranial ultrasound confirmed.
Postnatal Week 12	Multidisciplinary discharge planning completed.	Infant successfully discharged home with tracheostomy in situ, stable, pending future mandibular reconstruction.

## 3. Discussion

Before discussing the clinical findings, it is important to clarify the definition of success and failure within the EXIT‐to‐airway framework, as this distinction has been inconsistently applied in the literature. In this report, we distinguish between two levels of outcome: (1) success of the EXIT procedure, defined as the establishment of a functional airway under sustained uteroplacental circulation regardless of the method used, and (2) success of conventional intubation, defined as endotracheal intubation achieved via laryngoscopy or bronchoscopy. By this distinction, our case represents a successful EXIT procedure—the airway was secured via emergency tracheostomy under placental support, with stable neonatal hemodynamics and oxygenation—but a failure of conventional intubation due to extreme anatomic distortion. This aligns with Morris et al. [1], in which all three reported cases ultimately required tracheostomy and were nonetheless classified as successful outcomes.

This case represents the most severe end of the micrognathia–agnathia spectrum, where the near‐complete absence of the mandible, glossoptosis, and obliterated pharyngeal structures made conventional intubation essentially impossible [2, 4, 9, 10]. Our case occupies a distinct and uniquely valuable position within the existing EXIT literature. While larger case series frequently provide retrospective data regarding overall procedural survival, this report links specific, objective prenatal parameters (such as the extreme ultrasound IFA of 11° and MRI IFA of 40°) directly with intraoperative endoscopic documentation of a completely blind, landmark‐free oropharyngeal space. This rigorous correlation provides clinical teams with an explicit visual benchmark of what absolute conventional intubation failure looks like, highlighting that extreme micrognathia–agnathia is not merely a ‘difficult’ intubation scenario but can represent an anatomically impassable airway.

While the EXIT‐to‐airway procedure is specifically designed for such high‐risk situations, its success fundamentally relies on the existence of a functional airway lumen that can be accessed via laryngoscopy or bronchoscopy [1–3, 9, 10]. In our patient, the absence of an identifiable laryngeal inlet, even with rigid bronchoscopy, necessitated an immediate surgical airway despite ideal conditions and advanced team preparation. Morris et al. reported on a series of fetuses with severe micrognathia where the EXIT procedure, including tracheostomy under placental support, successfully established the airway [1]. Their work highlighted that the most severe cases—particularly those with a jaw index below the fifth percentile and marked polyhydramnios—are at the highest risk for failed conventional intubation and may require tracheostomy. Contemporary reviews of EXIT confirm that while overall airway success rates are high, failure still occurs in a small subset of cases with extreme anatomic derangement [2, 5, 9, 10]. Our case contributes to this limited subset by providing detailed prenatal imaging and intraoperative airway findings that align with a high risk for failure despite advanced tools. Accurate prenatal prediction of a difficult neonatal airway in fetuses with micrognathia remains a critical research focus [3, 4, 8]. Multiple ultrasound‐derived indices, such as the IFA, mandibular length, and the FNM angle, have been correlated with postnatal airway obstruction risk [4, 8]. Goergen et al. and Eyring et al. demonstrated that combining these biometric measurements with MRI‐derived assessment of tongue position and pharyngeal patency significantly improves the prediction of neonatal airway compromise and the need for advanced airway interventions [3, 4]. In our patient, the profoundly abnormal IFA and FNM angle, severe polyhydramnios, and MRI evidence of oropharyngeal–hypopharyngeal obliteration all pointed toward an exceptionally high probability of intubation failure.

The clinical decision‐making pathway during this procedure was governed by a strict, time‐sensitive algorithm focused on minimizing fetal hypoxia. In an EXIT framework, initial attempts at direct or video‐assisted visualization are standard to minimize the invasiveness of the airway approach. However, because the protective window of uteroplacental circulation is finite—usually beginning to degrade after 30–60 min due to uterine tone alterations and placental separation—the number of conservative maneuvers must be rigorously controlled [5, 9].

In this case, after three purposeful, gentle attempts using video laryngoscopy and rigid bronchoscopy, the complete absence of any anatomical markers or a visible glottis made it clear that further endoscopic manipulation would merely risk soft tissue trauma, hemorrhage, and futile time consumption. Consequently, the team strictly adhered to advanced multidisciplinary surgical airway algorithms, rapidly pivoting to emergency tracheostomy as the safest definitive method to protect the fetus from hypoxic–ischemic injury [10]. This tight operational coordination allowed the entire process—from hysterotomy to successful tracheal cannulation and cord clamping—to be completed in just 25 min, preserving neurological integrity as confirmed by subsequent normal cranial ultrasonography.

Beyond 2D imaging, 3D reconstruction of fetal airway anatomy from MRI datasets offers a more intuitive and comprehensive understanding of complex airway geometry [6–8]. Werner and colleagues demonstrated that MRI‐based virtual bronchoscopy can generate internal airway views in fetuses with cervical tumors and other obstructive lesions, accurately showing airway patency and thereby guiding perinatal management [6, 7]. Further work using plastic reconstruction and 3D models of fetal anatomy has illustrated how virtual views can be used to plan both surgical and airway strategies [7, 8]. Virtual fetal bronchoscopy can depict the luminal patency, the exact sites of narrowing, the relationship between the tongue and posterior pharyngeal wall, and, critically, the presence or absence of a visible laryngeal inlet. These features are highly relevant for predicting the feasibility of laryngoscopy and bronchoscopy during the EXIT procedure [6–8].

In the present case, neither 3D MRI airway reconstruction nor virtual fetal bronchoscopy was performed prenatally, as they were completely unavailable at our institution during the management of this patient, which precluded their use. As 3D MRI airway modeling and virtual bronchoscopy become more accessible, integrating these tools into EXIT planning algorithms for fetuses with severe micrognathia or agnathia appears both logical and necessary for optimizing perinatal outcomes.

## 4. Conclusion

The EXIT‐to‐airway procedure is an essential, life‐saving intervention for fetuses with anticipated airway obstruction, and its overall airway establishment success rate is high. However, success of the EXIT procedure must be distinguished from success of conventional endotracheal intubation: tracheostomy performed under placental support constitutes a successful EXIT outcome, not a failure. In cases of extreme mandibular deficiency with obliteration of the oropharynx and hypopharynx, conventional intubation will fail despite optimal conditions and advanced equipment, and primary tracheostomy must be anticipated.

This case highlights the importance of detailed prenatal imaging, including both ultrasound and MRI, and strongly supports the integration of 3D MRI airway reconstruction and virtual fetal bronchoscopy into the evaluation of fetuses with severe micrognathia/agnathia. Such advanced tools may significantly improve the prediction of conventional intubation feasibility, guide teams toward earlier preparation for primary tracheostomy, and ultimately enhance perinatal outcomes [1–8].

While it is crucial to recognize that management guidelines cannot be generalized globally based on a single clinical report, this case underscores the importance of rigorous multidisciplinary planning. It highlights that in the presence of complete absence of a functional airway channel at the oropharynx–hypopharynx and the lack of any identifiable laryngeal opening, strongly suggesting a near‐certain probability of failed endoscopic intubation, a planned primary tracheostomy under placental support should be considered as a primary strategic choice from the outset to minimize futile interventions and maximize neonatal safety.

## Funding

No funding was received for this research.

## Disclosure

All authors have read and approved the manuscript.

## Ethics Statement

The Institutional Review Board (IRB) of King Abdullah Specialized Children’s Hospital, Ministry of National Guard ‐ Health Affairs (MNGHA), Riyadh, Saudi Arabia, reviewed this study and determined it to be exempt from formal ethical approval as a retrospective single case report.

## Consent

The patient provided written informed consent for the publication of this case report and all accompanying clinical images. Because this is a retrospective observational case report of a single patient, clinical trial registration does not apply.

## Conflicts of Interest

The authors declare no conflicts of interest.

## Supporting Information

Additional supporting information can be found online in the Supporting Information section.

## Supporting information


**Supporting Information** CARE Checklist for difficulty airway case.

## Data Availability

Data used to support the findings of this study are available from the corresponding author upon reasonable request.

## References

[1] Morris L. M. , Lim F.-Y. , Elluru R. G. et al., Severe Micrognathia: Indications for EXIT-to- Airway, Fetal Diagnosis and Therapy. (2009) 26, no. 3, 162–166, 10.1159/000240162. 19776546

[2] De Jong R. and Fordham T. , Ex Utero Intrapartum Treatment (EXIT) Procedure, Statpearls, 2024, StatPearls Publishing. 38861624

[3] Goergen S. , Christie J. , Jackson T. et al., Predicting the Difficult Neonatal Airway in Fetuses with Micrognathia, Oropharyngeal or Neck Mass Lesions: Two‐Center Experience with Fetal MRI, Prenatal Diagnosis. (2024) 44, no. 13, 1593–1602, 10.1002/pd.6651. 39317943 PMC11628208

[4] Eyring J. B. , Allen W. P. , Bayazid L. O. , Hemeyer B. M. , Walker S. , and Orb Q. T. , Value of Imaging Measurements in Micrognathia‐Related Fetal Airway Obstruction Within a Fetal Center, The Laryngoscope. (2024) 135, no. 1, 393–401, 10.1002/lary.31747. 39239829 PMC11635131

[5] Gaffuri M. , Raffaeli G. , and Bullejos Garcia E. E. , The ex-utero Intrapartum Treatment Procedure: a Narrative Review, Frontiers in Pediatrics. (2025) 13, 10.3389/fped.2025.1601963. PMC12310721 40746351

[6] Werner H. , Dos Santos J. R. L. , Fontes R. , Daltro P. , Gasparetto E. , and Marchiori E. , Virtual Bronchoscopy in the Fetus, Ultrasound in Obstetrics and Gynecology. (2011) 37, no. 1, 113–115, 10.1002/uog.8886. 21182109

[7] Werner H. , Lopes J. , Tonni G. , and Araujo Junior E. , Plastic Reconstruction of Fetal Anatomy Using three-dimensional Ultrasound and Magnetic Resonance Imaging Scan Data in a Giant Cervical Teratoma. Case Report, Medical Ultrasonography. (2015) 17, no. 2, 252–255, 10.11152/mu.2013.2066.172.tert. 26052579

[8] Cang Z. , Cui J. , Pei J. et al., Prenatal Diagnosis of Micrognathia: a Systematic Review, Frontiers in Pediatrics. (2023) 11, 10.3389/fped.2023.1161421. PMC10130438 37124181

[9] Corroenne R. and Perrotin F. , Ex Utero Intrapartum Technique (EXIT): Indications, Procedure Methods and materno-fetal Complications-a Literature Review, Journal of Gynecology Obstetrics and Human Reproduction. (2022) 51, no. 1. 10.1016/j.jogoh.2021.102252 34638008

[10] Mohammad S. and Olutoye O. A. , Airway Management for Neonates Requiring Ex Utero Intrapartum Treatment (EXIT), Paediatric Anaesthesia. (2020) 30, no. 3, 248–256, 10.1111/pan.13818. 31898837

