# Supplementary material for: Extreme Airway Difficulty During EXIT in Severe Fetal Agnathia: Failed Conventional Intubation and Successful Emergency Tracheostomy—A Case Report
Source: Case Rep Anesthesiol. 2026 Aug 23;2026:9422230. doi: 10.1155/cria/9422230 (PMC13501007; doi:10.1155/cria/9422230)
Supplement: Supplementary file 1 [file CRIA-2026-9422230-s001.docx]

CARE Checklist of information to include when writing a case report

The diagnosis or intervention of primary focus followed by the words “case report”	**Page 1**

| Topic | Item |
| --- | --- |
| Title | 1 |
| Key Words | 2 |
| Abstract | 3a |
| (no references) | 3b |
|  | 3c |
|  | 3d |
| Introduction | 4 |
| Patient Information | 5a |
|  | 5b |
|  | 5c |
|  | 5d |
| Clinical Findings | 6 |
| Timeline | 7 |
| Diagnostic | 8a |
| Assessment | 8b |
|  | 8c |
|  | 8d |
| Therapeutic | 9a |
| Intervention | 9b |
|  | 9c |
| Follow-up and | 10a |
| Outcomes | 10b |
|  | 10c |
|  | 10d |
| Discussion | 11a |
|  | 11b |
|  | 11c |
|  | 11d |
| Patient Perspective | 12 |
| Informed Consent | 13 |

**Checklist item description**

**Reported on Line**

2 to 5 key words that identify diagnoses or interventions in this case report, including "case report"	**Page 2**

Introduction: What is unique about this case and what does it add to the scientific literature	**Page 2**

Main symptoms and/or important clinical findings	**Page 2**

The main diagnoses, therapeutic interventions, and outcomes	**Page 2**

Conclusion—What is the main “take-away” lesson(s) from this case? **Page 2**

One or two paragraphs summarizing why this case is unique (may include references)	Page 3

De-identified patient specific information	**Page 3 - 4**

Primary concerns and symptoms of the patient	**Page 3 - 4**

Medical, family, and psycho-social history including relevant genetic information **Page 3 - 4**

Relevant past interventions with outcomes	**-**

Describe significant physical examination (PE) and important clinical findings **Page 3 - 4**

Historical and current information from this episode of care organized as a timeline	**Page 5**

Diagnostic testing (such as PE, laboratory testing, imaging, surveys) **Page 3 - 4**

Diagnostic challenges (such as access to testing, financial, or cultural)	**Page 10**

Diagnosis (including other diagnoses considered)	**Page 3 - 4**

Prognosis (such as staging in oncology) where applicable	**Page 3 - 4**

Types of therapeutic intervention (such as pharmacologic, surgical, preventive, self-care)	**Page 4**

Administration of therapeutic intervention (such as dosage, strength, duration)	**Page 4**

Changes in therapeutic intervention (with rationale	**Page 4**

Clinician and patient-assessed outcomes (if available)	**Page 4**

Important follow-up diagnostic and other test results	**Page 4**

Intervention adherence and tolerability (How was this assessed?) **Page 4**

Adverse and unanticipated events	**Page 4**

A scientific discussion of the strengths AND limitations associated with this case report **Page 8 – 9 - 10**

Discussion of the relevant medical literature with references	Page 8 – 9 - 10

The scientific rationale for any conclusions (including assessment of possible causes) **Page 9 - 10**

The primary “take-away” lessons of this case report (without references) in a one paragraph conclusion **Page 10 - 11**

The patient should share their perspective in one to two paragraphs on the treatment(s) they received	**-**

Did the patient give informed consent? Please provide if requested **The Family**

Yes	No □
